# Molecule-to-Material-to-Bio Nanoarchitectonics with Biomedical Fullerene Nanoparticles

**DOI:** 10.3390/ma15155404

**Published:** 2022-08-05

**Authors:** Xuechen Shen, Jingwen Song, Kohsaku Kawakami, Katsuhiko Ariga

**Affiliations:** 1 Graduate School of Frontier Sciences, The University of Tokyo, 5-1-5 Kashiwanoha, Kashiwa 277-8561, Chiba, Japan; 2 Research Center for Functional Materials, National Institute for Materials Science (NIMS), 1-1 Namiki, Tsukuba 305-0044, Ibaraki, Japan; 3 Graduate School of Pure and Applied Sciences, University of Tsukuba, 1-1-1 Tennodai, Tsukuba 305-8577, Ibaraki, Japan; 4 WPI Research Center for Materials Nanoarchitectonics (MANA), National Institute for Materials Science (NIMS), 1-1 Namiki, Tsukuba 305-0044, Ibaraki, Japan

**Keywords:** assembly, biomedical application, fullerene, nanoarchitectonics, nanoparticle

## Abstract

Nanoarchitectonics integrates nanotechnology with various other fields, with the goal of creating functional material systems from nanoscale units such as atoms, molecules, and nanomaterials. The concept bears strong similarities to the processes and functions seen in biological systems. Therefore, it is natural for materials designed through nanoarchitectonics to truly shine in bio-related applications. In this review, we present an overview of recent work exemplifying how nanoarchitectonics relates to biology and how it is being applied in biomedical research. First, we present nanoscale interactions being studied in basic biology and how they parallel nanoarchitectonics concepts. Then, we overview the state-of-the-art in biomedical applications pursuant to the nanoarchitectonics framework. On this basis, we take a deep dive into a particular building-block material frequently seen in nanoarchitectonics approaches: fullerene. We take a closer look at recent research on fullerene nanoparticles, paying special attention to biomedical applications in biosensing, gene delivery, and radical scavenging. With these subjects, we aim to illustrate the power of nanomaterials and biomimetic nanoarchitectonics when applied to bio-related applications, and we offer some considerations for future perspectives.

## 1. Introduction: Nanoarchitectonics for Molecules, Materials, and Biofunctions

Development of human society is keenly dependent on the development of materials. It is no exaggeration to say that it is the science of materials that empowers our modern lifestyle. Currently, the solution to our problems in the energy [1,2,3,4], environmental [5,6,7,8], and medical fields [9,10,11,12] lies in the development of new functional materials. The development of materials is supported by an array of well-established scientific fields. These include organic chemistry [13,14,15,16], inorganic chemistry [17,18,19,20], coordination chemistry [21,22,23], polymer chemistry [24,25,26,27], supramolecular chemistry [28,29,30,31], biochemistry [32,33,34,35], and other materials sciences [36,37,38,39]. These fields have produced a variety of high-performance materials. It has also become clear that control over structure at a finer scale is important for higher functionality.

This trend has been accelerating with the advancement of nanotechnology. For example, advancement of electron microscopy and other technologies has elucidated the behavior, assembly, and reactions of molecules and materials at very high resolution [40,41,42]. As seen in a recent review paper by Harano, advanced electron microscopy technologies enable us to observe unitary processes in chemical reactions and material nucleation [43]. The development of new techniques, such as probe microscopy, provides very high magnification of molecular motion and structures on solid surfaces [44,45]. It also makes it possible to see reaction products on the surface in situ. For example, Müllen, Narita, and coworkers demonstrated high-resolution observation with noncontact atomic force microscopy of molecular-level reactions on a solid surface [46,47]. It also makes it possible to evaluate the reaction behavior of a single molecule sandwiched between the substrate and the probe. Kazuma reported real-space evaluations of plasmon-induced dissociation reactions using scanning tunneling microscopy [48]. It also opens the new field of probe-based chemical reactions. Kawai and coworkers have developed local probe chemistry for molecularly site-specific reactions using a tiny probe tip [49,50]. Thus, advances in nanotechnology have enabled a high level of observation, characterization, and manipulation at the atomic, molecular, and nanoscale. Nanotechnology is not only improving our understanding of nanoscale phenomena, but it is also contributing in no small measure to material synthesis.

**Figure 1:**
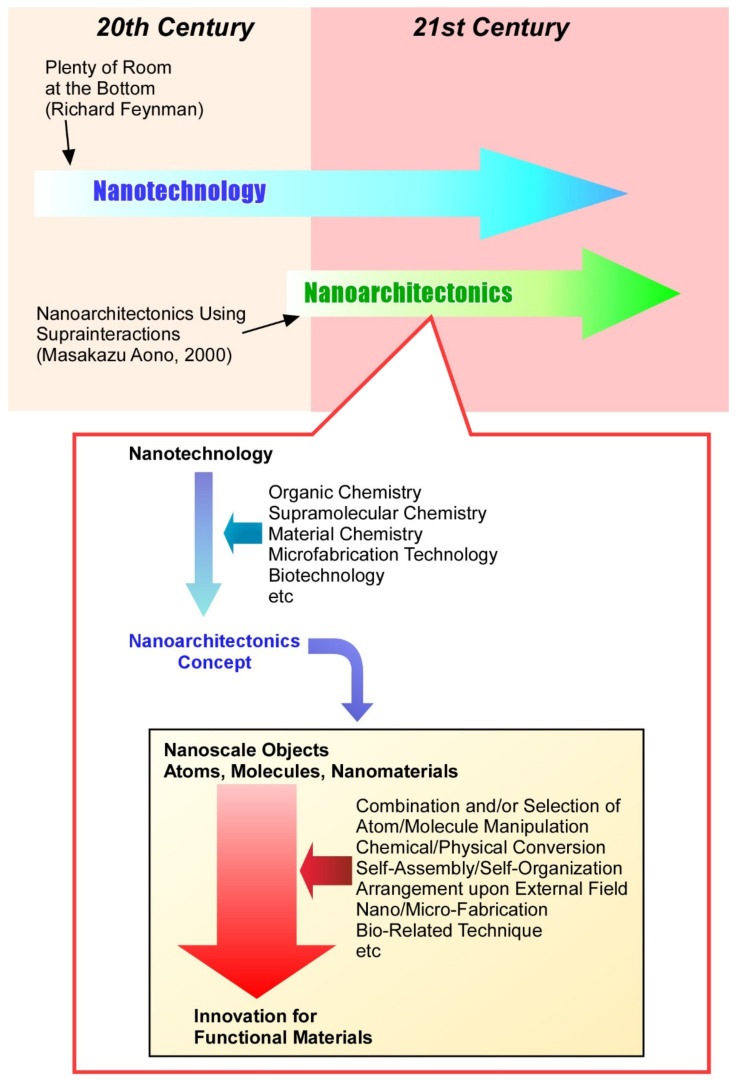
Outline of nanoarchitectonics concept to create functional material systems from nanoscale units such as atoms, molecules, and nanomaterials through combination and/or selection of atom/molecule manipulation, chemical/physical conversion, self-assembly/self-organization, arrangement on external fields, nano/micro-fabrication, and bio-related techniques.

As nanotechnology advances, our understanding of nanoscale phenomena has deepened, and the creation of functional materials and systems has entered a new phase. In order to further promote such developments, it is necessary to propose a concept to represent them. Just as Richard Feynman proposed nanotechnology [51,52], Masakazu Aono established the concept of nanoarchitectonics (Figure 1) [53,54]. Nanoarchitectonics can be regarded as a post-nanotechnology concept [55]. Nanoarchitectonics integrates nanotechnology with organic chemistry, supramolecular chemistry, material chemistry, microfabrication technology, and biotechnology to create functional material systems from nanoscale units such as atoms, molecules, and nanomaterials [56,57]. More specifically, it can be achieved through a mix-and-match of atom/molecule manipulation, chemical/physical conversion, self-assembly/self-organization, arrangement through external fields, nano/micro-fabrication, and bio-related techniques to build functional materials and systems from nano-units [58,59].

Since this methodology is based on a highly general concept, there are almost no restrictions on the materials and functions for which it can be applied. Therefore, nanoarchitectonics is being used in many research fields: basic science fields such as materials production [60,61,62], structural regulation [63,64,65], investigation of physical science [66,67,68], and basic biochemistry [69,70,71]; application-oriented fields such as energy [72,73,74], environmental [75,76,77], and biomedical [78,79,80]; and target-oriented fields such as catalysts [81,82,83], sensors [84,85,86], devices [87,88,89], and drug-delivery systems [90,91,92]. Since materials are fundamentally composed of atoms and molecules, the nanoarchitectonics methodology of building materials from atomic and molecular nano-units may be applicable to all materials. Analogous to the Theory of Everything in physics [93], nanoarchitectonics may be regarded as the Method for Everything in materials science [94].

Nanoarchitectonics shares several similarities with the formation of functional structures and their roles in biological systems. In nanoarchitectonics approaches, various elementary processes are often combined to form functional structures. In some cases, functional material structures are often created by combining self-assembly [95,96,97], which is mediated by equilibrium processes, and Langmuir–Blodgett (LB) [98,99,100,101] or layer-by-layer (LbL) assembly [102,103,104,105], which are nonequilibrium processes involving human manipulation. For example, mesoporous materials synthesized via self-assembly and template synthesis are made multilayered through LbL to develop sensing materials [106,107] and drug delivery systems [108]. Functional structures created in this way often have a hierarchical structure. Therefore, structure formation via nanoarchitectonics can be considered effective for creating hierarchical structures [109]. Considering that the expression of high functionality through hierarchical structuring is universally observed in biofunctional systems, similar functionality may prove achievable with nanoarchitectonics. In living organisms, proteins with various functions and the chromophores contained in them form a rational hierarchical structure to achieve unidirectional information transfer, efficient energy conversion and harvesting, and continuous material conversion [110,111]. The formation of hierarchical functional structures through nanoarchitectonics has the potential to produce such advanced functions.

Another similarity between nanoarchitectonics and biofunctional systems is functional synergy [112,113], which derives from the behavior of nanoscale objects and interactions between molecules and materials. Phenomena at the nanoscale are often accompanied by uncertainties such as thermal fluctuations, statical distributions, and quantum effects. Since nanoarchitectonics assembles unit structures with these uncertainties, the effect is likely to be synergistic as opposed to simply their summation. In fact, such characteristics are also universally observed in biological functions. In biological systems, proteins move in thermal fluctuations and function with a certain degree of uncertainty [114,115]. Yet when they are assembled and synergized, they exhibit surprisingly high functionality. This is quintessentially different from artificial devices, which are made by a series of prescribed mechanisms with high accuracies. In contrast, the functions of biological systems result in soft, ambiguous, yet highly intelligent outputs. Such bio-like functional outputs are likely to be manifested in the formation of functional material systems through nanoarchitectonics [116].

Considering the characteristics of nanoarchitectonics as described above, nanoarchitectonics may fully demonstrate its capabilities when applied to biological systems. The ultimate goal of nanoarchitectonics is to build intelligent functional material systems, such as biological systems, from atoms, molecules, and nanomaterials [117,118]. Although such goals are far from realization, it is worthwhile to discuss the relationship between nanoarchitectonics and bio-related applications [119,120]. In this review, bio-related applications of fullerene nanoparticles, in particular, will be discussed as an example. We will explain how fullerenes—zero-dimensional molecules composed of a single element—become nanoparticle-like materials and how they interact with biosystems. We introduce the topic as an example of molecule-to-material-to-bio nanoarchitectonics.

**Figure 2:**
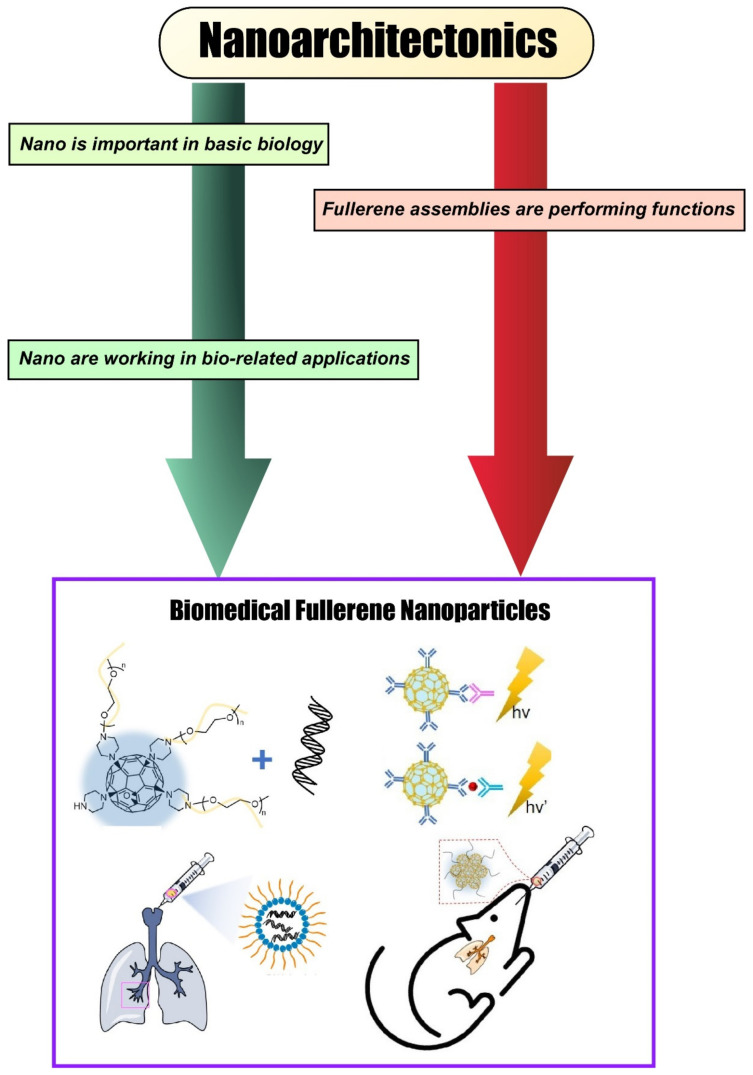
Outline and story flow of this review article. First, we present nanoscale interactions in biology and bio-related applications, giving an overview of how they fit into the nanoarchitectonics framework for materials design and synthesis. Then, we take a deep dive into a particular building-block material frequently seen in nanoarchitectonics approaches: fullerene; we take a closer look at recent research on fullerene nanoparticles, paying special attention to biomedical applications in biosensing, gene delivery, and radical scavenging.

As shown in Figure 2, this review article does not simply introduce the main topic, but it also discusses the relevant background in its first sections: (i) nano in basic biology, (ii) nano in bio-related applications, and (iii) fullerene assemblies performing functions. In other words, we will outline how nanostructures and their assemblies are used in basic biology and in bio-related applications and how assemblies of simple molecules such as fullerenes can perform functions, focusing on recent examples. Upon this background, biomedical fullerene nanoparticles will be introduced as the main topic. It should be noted that the examples given in this paper are chosen to illustrate their characteristics and are not exhaustive. Through such examples and discussions, the importance of molecule-to-material-to-bio nanoarchitectonics will be explained.

## 2. Nano in Basic Biology

**Figure 3:**
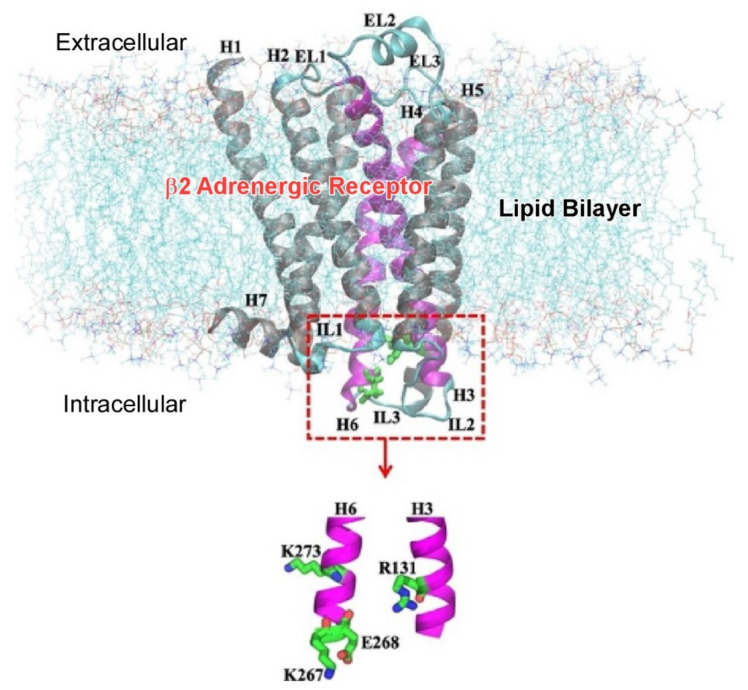
A β2 adrenergic receptor in a lipid bilayer composed of palmoyloleoylphosphatidylcholine for molecular dynamics simulation. Reprinted with permission from Reference [122]. Copyright 2021, Chemical Society of Japan.

In biological systems, functional molecules such as proteins operate delicately under thermal fluctuations. They are arranged in exquisite positional relationships and cooperate with each other to perform a function. These characteristics of biological systems give nice intuitions for material design in nanoarchitectonics. Analyzing and understanding biological systems may inform a strong basis for nanoarchitectonics. Research into biological functions is being conducted from various perspectives. Molecular dynamics simulations of protein behavior are key to elucidating relevant biological functions. Morita et al. developed a method called parallel cascade selection molecular dynamics. They successfully applied it to the folding of Chignolin to efficiently identify multiple metastable states, including intermediate, misfolded, and native states [121]. Such delicate behavior of a protein also depends on the nanoarchitected environment. For example, the lipid bilayer has a major influence on the structure and dynamics of membrane proteins. Mahmood and Yamashita showed a β2 adrenergic receptor in a lipid bilayer composed of palmoyloleoylphosphatidylcholine (Figure 3) using molecular dynamics simulation [122]. Interestingly, they found that the lipid force field significantly affects the conformation of the protein, even though the sites involved in protein activation are located far from the lipid. This difference may be attributed to the modified interaction energies between the protein and the lipid bilayer. This is an example of the subtle nanoarchitectonics of lipids and proteins reflected in functional expression.

The organization of proteins and dyes nanoarchitectonically arranged in biological systems and their interactions have also been the subject of interesting studies. Takahashi, Ohshima, and coworkers investigated the excitonic transitions and dimeric composition of native water-soluble chlorophyll protein CP633 using absorption and circular dichroism spectra [123]. Calculation of dimer composition based on the spectral results suggests that CP663 preferentially accommodates chlorophyll homodimers over the corresponding heterodimer. Mishima, Shoji, and coworkers theoretically investigated behaviors of the chromophore in C-phycocyanin and phycocyanobilin using time-dependent density functional theory and natural bond orbital methods [124]. Geometric analyses of bond lengths, interatomic angles, and dihedral angles reveal that the intermolecular interactions of the propionic acid sidechain play an important role in determining the excited-state molecular structure. These results indicate that the absorption spectra and excited-state structures of phycocyanobilin are nanoarchitectonically organized during natural photosynthetic processes.

Functional bioproteins are also being studied. Rhodopsins, also called retinal proteins, are quintessential photoreceptive proteins. Their photoreactions in nanoarchitectonics structures have been the focus of much attention in physics, chemistry, and biology. For example, a recent review article by Kandori outlines historical aspects and recent advances in retinal protein research [125]. The molecular mechanism of bacteriorhodopsin, a light-driven H^+^ pump and the best-studied microbial rhodopsin, is described in the review paper. The molecular properties and several variations of channelrhodopsin, a light-gated ion channel, are also discussed. Understanding the molecular mechanism of rhodopsin is a prerequisite for future functional design of useful optogenetics tools. As another quintessential protein, cytochrome c is known as a multifunctional, water-soluble heme protein. The review article by Hirota and Nagao describes newly discovered aspects of cytochrome c, including three-dimensional domain swapping, peroxidase activity, membrane interaction, and Met80 sulfoxide modification [126]. In the mitochondrial respiratory chain, it transfers electrons from the cytochrome bc1 complex (complex III) to the cytochrome c oxidase complex (complex IV) and may also trigger apoptosis. Cytochrome c molecules can form oligomers and polymers through three-dimensional domain swapping during which the C-terminal α-helix is exchanged. Although the exchange regions differ, three-dimensional domain swapping is suspected to be a common feature of c-type cells. This can occur during protein folding and expression in the cell. Stereoisomerization dissociates Met80 from heme iron, resulting in decreased electron transfer capacity of cytochrome c, but increased peroxidase activity. High peroxidase activity may promote lipid oxidation and ultimately induce apoptosis. These new findings not only help us understand the nanoarchitectonics structure–function relationship of the multifunctional cytochrome c, but also demonstrate that well-studied proteins may have yet unknown properties.

**Figure 4:**
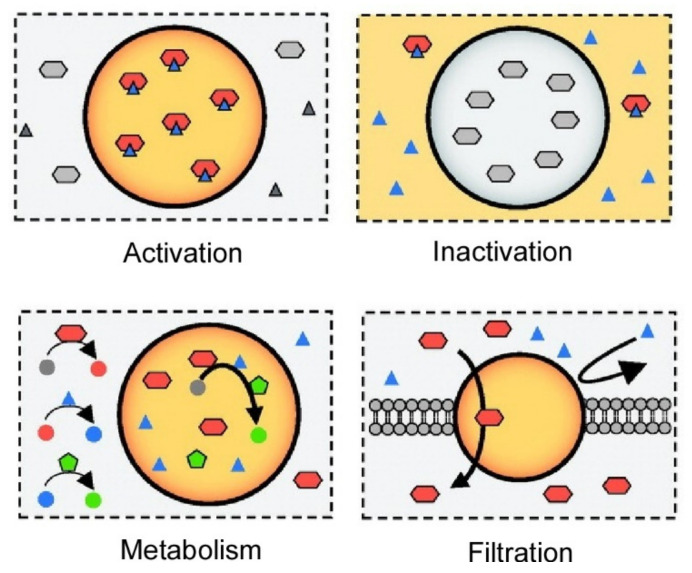
Phase-separated droplets composed of biomolecules for a variety of intrinsic or synthetic functions such as activation, inactivation, metabolism, and filtration. Reprinted with permission from Reference [127]. Copyright 2021, Chemical Society of Japan.

The effects of large structural changes such as phase separation on biological functions have also been studied. As described in a recent review by Kamimura and Kanai [127], liquid–liquid phase separation in living organisms has recently emerged as a biological principle that has the potential to significantly change the current perception of cellular systems. Biomolecules such as various proteins and RNAs undergo liquid–liquid phase separation to perform various cellular functions. Phase-separated droplets composed of biomolecules, also called biomolecular condensates, perform a variety of intrinsic or synthetic functions, such as activating or inactivating specific molecules, buffering intracellular molecular concentrations, sensing stimuli, compartmentalizing, promoting sequential reactions, and filtering molecules (Figure 4). Biomolecules in droplets can be specifically activated by activating molecules or their substrates. Conversely, biomolecules in the droplet can be specifically inactivated by the exclusion of activating molecules or substrates from the droplet. Enzymes involved in metabolic pathways can be specifically concentrated in the droplet to promote continuous reactions. The droplet on the cell surface acts as a filter for molecules, allowing selective transport of specific molecules into the cell. On the other hand, dysfunction of liquid–liquid phase separation has been associated with a variety of diseases, including neurodegenerative diseases and cancer. Thus, biomolecular condensates are important considerations in chemical approaches such as drug discovery and chemical biology tools. This emerging field can bridge biology and physical chemistry. Moreover, the introduction of nanoarchitectonics would be useful.

As described above, basic research in various biological systems relies heavily on nano-level structural control and its dynamics. Therefore, the introduction of nanoarchitectonics into these fields may unify artificially segregated research efforts into a scientific paradigm with common concepts.

## 3. Nano in Bio-Related Applications

In the previous section, we briefly discussed the importance of considering nanostructures and their dynamics in basic biological research. In this section, we present recent examples demonstrating the importance of controlling nanostructures in more application-oriented fields such as biohazard detection and various therapies. We also forecast potential contributions of nanoarchitectonics in the biomedical field.

Currently, COVID-19 is spreading throughout the world, causing tremendous damage to health, economies, and livelihoods. Although much research has been conducted in medical institutions and hospitals, there have been relatively few attempts to solve this pressing problem through chemical approaches and nanotechnological considerations. A recent review article by Komiyama [128] provides an overview of the chemical information of COVID-19, with particular emphasis on understanding it at the molecular level. The conformational change of the spike protein is critically important for the virus to invade human cells. Furthermore, the role of SARS-CoV-2 mutations in promoting virulence is discussed, primarily in terms of the interaction between the spike proteins and the receptors on human cells (ACE2). Finally, an origin for the unprecedentedly high virulence of this virus is proposed. All of these illustrate the importance of chemical nanoarchitectonics in COVID-19. Watanabe et al. calculated the interaction energies between the receptor binding domain of the SARS-CoV-2 spike protein and neutralizing antibodies using the fragment molecular orbital method [129]. A mutation was thought to be involved in escape from the antibody. Ito and coworkers developed a SARS-CoV-2 protein-specific automated microarray diagnostic system using photoimmobilized viral proteins [130]. The photoimmobilization strategy immobilizes proteins through a crosslinking reaction of photoreactive polymers. An aqueous solution of the protein is spotted onto a polymer-coated plate and dried in air. The proteins are then immobilized by irradiation from a UV lamp. Microarrays fabricated via nanoarchitectonics using microfabrication techniques allow multiple assays to be performed on small amounts of patient serum for different viral proteins. The assays are faster than conventional nitrocellulose microarrays and more sensitive than immune chromatography.

**Figure 5:**
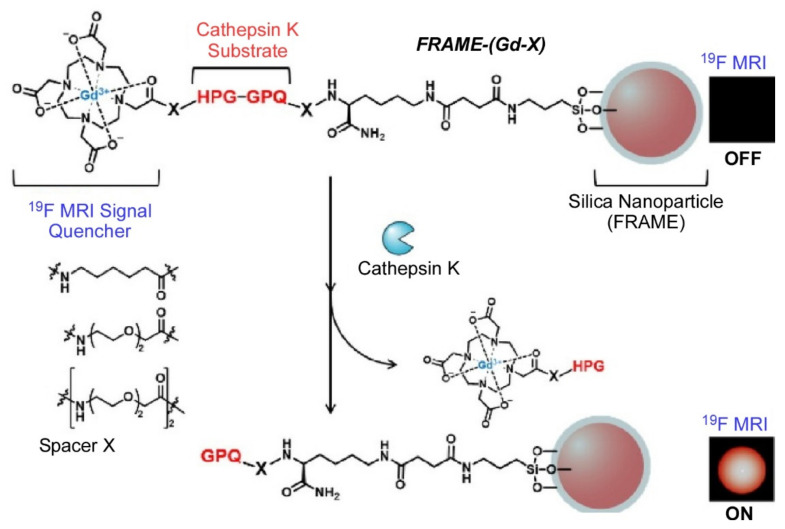
A novel ^19^F-MRI probe, FLAME-(Gd-X), for the detection of Cathepsin K. The ^19^F-NMR signal intensity of these probes is suppressed by paramagnetic relaxation due to the Gd^3+^ complex, but signal intensity increases specifically upon cleavage of the substrate by Cathepsin K. Reprinted with permission from [132]. Copyright 2021, Chemical Society of Japan.

Detection of hazardous biofactors is also being investigated through structural control via nanoarchitectonics. For example, microcystins are a type of toxin produced mainly by cyanobacteria; in particular, microcystin-leucine-arginine (Microcystin-LR) is one of the most-toxic freshwater toxins and is associated with many accidents and endangering human health. Therefore, it is very important to detect microcystin-LR in drinking water and environmental water samples. In their recent review article [131], Pang, Yang, and coworkers described the current state-of-the-art microcystin-LR biosensing platforms. The advantages and limitations of quintessential transduction techniques were evaluated to identify the most-efficient detection system for potentially harmful cyanobacteria. Other biofactor risks considered include the detection of Cathepsin K. Cathepsin K is a protease that is expressed in osteoclasts and degrades bone tissue such as type I collagen fibers. Overexpression of Cathepsin K has been implicated in osteoporosis, rheumatoid arthritis, and bone metastasis. Therefore, detection of Cathepsin K activity is important for elucidating the mechanisms of these diseases and developing new drugs. Kikuchi and coworkers developed a novel ^19^F-MRI probe for the detection of Cathepsin K (Figure 5) [132]. In their FLAME-(Gd-X) probe, the Gd^3+^ complex is affixed on the surface of perfluorocarbon-encapsulated silica particles via a Cathepsin K substrate and three different hydrophobic/hydrophilic linkers. FLAME contains a large number of liquid perfluoro-15-crown-5-ether molecules with the equivalent of 20 fluorine atoms in their cores, and it shows a strong ^19^F-NMR signal. Furthermore, by covering the surface with silica, the nanoparticles have high stability against aqueous solutions and organic solvents. The ^19^F-NMR signal intensity of these probes is suppressed by paramagnetic relaxation due to the Gd^3+^ complex, but signal intensity increases specifically upon cleavage of the substrate by Cathepsin K. The prepared ^19^F-MRI probe based on paramagnetic relaxation may have potential application for in vivo detection of Cathepsin K activity.

**Figure 6:**
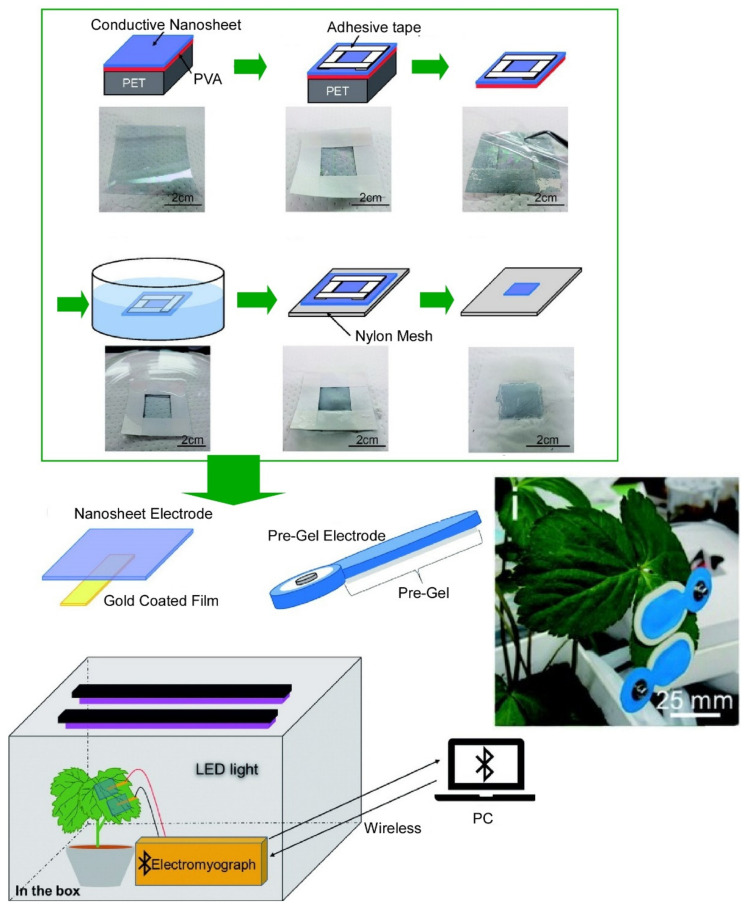
Conductive nanosheets used as bioelectrodes to measure bioelectrical potential of plant leaves wirelessly for up to approximately 1500 h. Reprinted with permission from Reference [134]. Copyright 2020, Chemical Society of Japan.

Supramolecular assembly nanoarchitectures, which are recurrent players in nanoarchitectonics research, are useful for the development of chemical and biochemical sensors. In a recent review article [133], Minami presented a nanoarchitectonics-type methodology that combines bottom-up and top-down approaches for the development of sensors that fully meet requirements for practical use. The self-assembled sensors amplify light response patterns due to their reversible characteristics. Furthermore, the combination of chemosensor arrays and chemometrics, such as machine learning, enabled accurate qualitative and quantitative measurements of real samples. Fujie and coworkers demonstrated that freestanding conductive polymer nanosheets (conductive nanosheets) can be used as bioelectrodes in plant leaves [134]. The ultra-thin and lightweight structure of the conductive nanosheets gives them ultra-compatibility with and superb physical adhesion to uneven surfaces, such as plant leaf veins, without the use of chemical adhesives. The nanosheet electrode for plant-leaf bioelectrical potential measurement is fabricated with a three-layer structure consisting of a conductive nanosheet, a water-soluble poly(vinyl alcohol) support layer, and a poly(ethylene terephthalate) film, which is attached to the plant leaf through a peeling and transfer process (Figure 6). Combining this conductive nanosheet with a Bluetooth system, the bioelectrical potential of plant leaves can be measured wirelessly for up to approximately 1500 h. Minimally invasive measurements using conductive nanosheets could pave the way for innovative methods to analyze plant bioactivity in agricultural and food-science applications. As another sensing mechanism, nanoplasmonic biosensors based on localized surface plasmon resonance have attracted much attention due to their rapid detection and label-free functionality. Zhu, Tamiya, and coworkers fabricated a cauliflower-like nanopillar structure based on a cyclo-olefin polymer material to improve sensitivity based on the hot-spot-effect theory [135]. Oxygen plasma etching was applied during the chip fabrication process to engrave cauliflower-like nanostructures onto the surface of nanopillars fabricated with nanoimprint lithography. They confirmed that this sensor chip is more sensitive than the untreated nanopillar structure chip, which exemplifies improved biosensor performance by structural design using nanoarchitectonics.

Biomedical applications of nanomaterials are also flourishing. Bioactive systems incorporating nanomaterials can be assembled via nanoarchitectonics. For example, in the past few decades, two-dimensional nanomaterials have been investigated in various bio fields, such as drug delivery systems and diagnostic/imaging materials [136,137,138]. In particular, layered double salts, layered rare-earth hydroxides, and layered double hydroxides have been the focus of much investigation. Some two-dimensional nanomaterials have been used as drug-delivery vehicles due to their low toxicity, high solubility in body fluids, high tumor-targeting efficiency, and high drug-loading capacity. Choy and coworkers summarized recent progress in diagnostic and imaging applications of these two-dimensional nanomaterials [139]. Two-dimensional nanoparticles have applications in optical imaging, magnetic resonance imaging, single-photon-emission computed tomography, positron-emission tomography, and computed tomography. These approaches are further used for molecular imaging in image-guided therapy and precision therapy. Therapeutic effects can be enhanced by applying external stimuli to nanomaterials. As described in a recent review article by Paris and Vallet-Regí [140], ultrasound has recently attracted attention as an external stimulus that can activate various types of nanomaterials and can be applied therapeutically. One of the features that makes ultrasound particularly attractive as a triggering stimulus for nanomedicine is that it can be applied in a noninvasive, deep-focused manner to the body. The combination of ultrasound and nanoparticles can produce a variety of biological effects, including inducing drug release, modifying nanoparticle biodistribution, producing ultrasound-derived biological effects, and developing theranostic agents.

Polymer self-assembly is used as a nanoarchitectonics approach to create nanostructures for biomedical applications. The fabrication of biocompatible polymer carriers for sustained and controlled drug delivery has been extensively studied for many years [141,142]. Furthermore, systems based on naturally occurring polymers have outperformed conventional polymers in biocompatibility, biodegradability, and cost-effectiveness. Polysaccharides are among the most common biopolymers found in nature and can achieve a high degree of complexity and excellent biological properties. In recent work by Rodrigues, Mano, and coworkers, they proposed the use of biodegradable and biocompatible microcarriers synthesized from laminarin, which has biological activity such as antibacterial effects [143]. When incubated with human adipose stem cells and the L929 cell line, little cytotoxicity was observed, and no membrane or nuclear damage was detected. Thus, the microparticles synthesized from laminarin proved to be a cost-effective, biocompatible and biodegradable nanoarchitectonics system.

**Figure 7:**
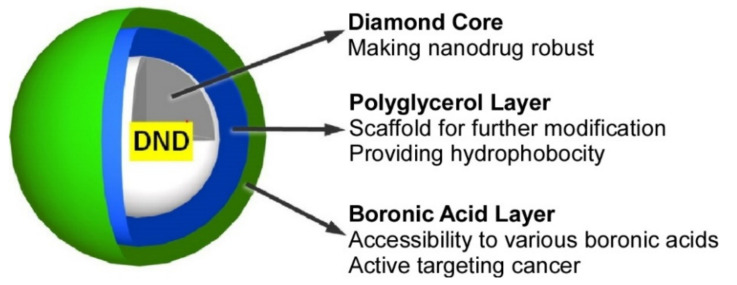
Boronic-acid-functionalized detonation nanodiamonds as an anticancer agent for boron neutron capture therapy. Reprinted with permission from [144]. Copyright 2021, Chemical Society of Japan.

Detonation nanodiamonds have attracted attention, especially in the field of nanomedicine, because of their biocompatibility and various functionalities through surface modification. On the other hand, boron neutron capture therapy is an advanced cancer therapy using a ^10^B fission reaction induced by neutron irradiation. Recently, selective and retentive delivery of ^10^B to cancer tissues using boron-containing nanoparticles has been investigated. Nishikawa, Komatsu, and coworkers investigated boronic acid-functionalized detonation nanodiamonds as an anticancer agent for boron neutron capture therapy [144]. Polyglycerol-modified detonation nanodiamonds were introduced with a phenylboronic acid moiety through a multistep organic transformation (Figure 7). The process is scalable and reliable due to simple covalent chemistry, and the resulting product is well-dispersed and chemically and physically stable under physiological conditions. In in vivo experiments, the resulting material accumulated in tumors and was found to exert a boron neutron capture therapy effect upon neutron irradiation. These results indicate that phenylboronic-acid-functionalized detonation nanodiamonds are promising candidates as anticancer nanodrugs for boron neutron capture therapy.

## 4. Fullerene Assemblies Performing Functions

In the previous sections, we explained the importance of nanoarchitectonics, a new paradigm of materials science based on nanotechnology. The importance of nanostructural control in basic biology and biomedical research were reviewed with recent examples. In this section, we overview the categories of assembled structures that can be formed from simple molecules and their associated functions. In the following section, we use fullerene molecules (C_60_, C_70_, etc.) to set up a deep dive into biomedical fullerene nanoparticles. A fullerene molecule is a spherical, zero-dimensional carbon allotrope. As a component used in nanoarchitectonics, it is unitary and very fundamental. At the same time, molecular interactions of fullerene molecules give rise to various functions due to their electronic properties and hydrophobic nature [145,146]. In this section, some examples of structures and functions of fullerenes and derivatives in nanoarchitectonics are overviewed as selected from recent research.

Attempts to create relatively small nanocarbons, such as fullerene analogues and nanographene, via organic synthesis have been reported [147,148,149]. This may represent the foundational step of nanocarbon-based nanoarchitectonics. For example, a recent review article by Ikemoto and Isobe [150] describes the synthesis of polygonal networks forming nanometer-sized curved organic π-conjugate molecules using the planar triangular structure of 1,3,5-trisubstituted benzene, named phenine, as the basic unit. A series of techniques can be used to synthesize large carbonaceous molecules reaching up to 4 kDa. The unique structural and electronic features of the synthesized defective nanocarbon molecules, i.e., geodesic phenine frameworks, are also discussed. Harano, Nakamura, and coworkers reported some sophisticated observations of fullerene interactions [151]. Molecular observations are usually made as an average of many molecules or on molecules immobilized (or motion-restricted) on a solid surface. Observation of molecules that are relatively free to move is usually difficult. Mechanical motion at the level of single molecules is subject to quantum effects and is influenced by fluctuations in the environment. Such behavior is the essence of nanoarchitectonics, a phenomenon concerned with the synergy of function and structure arising from fluctuations. As enablers of such observations, they reported in situ observations of shuttle motion, rotation, and interaction of fullerene molecules incorporated in carbon nanotubes with sub-millisecond precision using electron microscopy, high-speed cameras, and noise-reduction algorithms. The mechanical motion of fullerene molecules coupled with the vibration of carbon nanotubes was found to be nonlinear and stochastic, with rich molecular dynamics that are often nonreproducible. They elucidated the work–energy relationship at the molecular level, which cannot be detected by time-averaged measurements or microscopy using conventional techniques.

**Figure 8:**
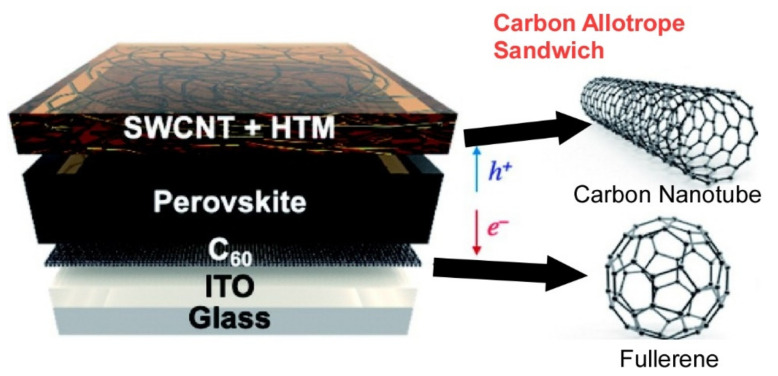
Perovskite solar cell fabricated by sandwiching a perovskite layer between two carbon allotropes: C_60_ and carbon nanotubes. Reprinted with permission from [152]. Copyright 2021, Chemical Society of Japan.

Fullerenes and their assemblies are widely used in various applications. As an example of utilizing the electronic properties of fullerenes, Matsuo describes the use of fullerene derivatives, endohedral fullerenes, and nanocarbon nanotubes such as carbon nanotubes to improve the power-conversion efficiency and stability of organic and perovskite solar cells [152]. If the fullerene derivatives are properly designed, they can function as an electron transport layer via passivation of defects at the interface between the perovskite crystal and the inorganic charge-selective layer. A perovskite solar cell was fabricated by sandwiching a perovskite layer between two carbon allotropes, C_60_ and carbon nanotubes, as shown in Figure 8. As a result, high stability, excellent hysteresis properties, and high conversion efficiency were obtained. Lithium-ion endohedral fullerenes can be doped with organic semiconductor molecules or carbon nanotubes to improve not only power-conversion efficiency, but also stability, as neutral lithium endohedral fullerenes are formed to eliminate oxygen. As an application of the hydrophobic and aromatic properties of nanocarbons, Otsuka et al. discussed the application of nanocarbon materials for separation functions [153]. In recent decades, the development of various new separation media has greatly advanced separation technology, especially nanocarbon materials such as graphene, carbon nanotubes, and fullerenes, which have recently been applied for effective separation and sensitive detection. In their recent review article, they summarize the basic preparation protocols of new separation media consisting of nanocarbon materials and their many applications.

**Figure 9:**
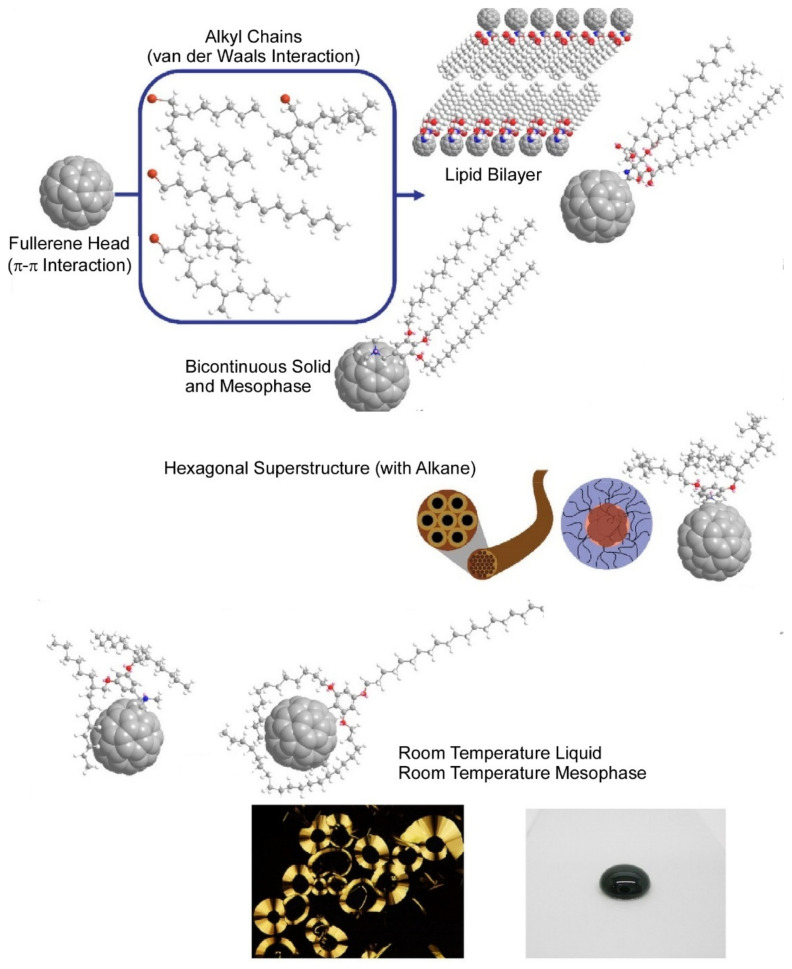
Diverse assembly structures formed by alkylated fullerenes. Reprinted with permission from [154]. Copyright 2021, Chemical Society of Japan.

There have also been many reports on derivatives of fullerenes. Neal and Nakanishi et al. reported on the diverse assembly structures formed by alkylated fullerenes [154]. The photophysical and electronic properties of fullerenes (C_60_) have been extensively studied and have proven useful in the fabrication of various devices. In some cases, processing them into device structures may be problematic. By simply attaching alkyl sidechains, this highly crystalline solid can be converted to alkyl-C_60_ hydrophobic amphiphiles for easier processing. Alkyl-fullerene materials form hydrophobic amphiphiles in which π–π interactions between fullerenes and van der Waals forces between alkyl units are dominant. Chain selection leads to a wide range of more exotic but unpredictable structures. The formation of lipid films, crystalline nanostructures, mesophases, and even liquid phases at room temperature become possible. Some of these are kinetically controlled by precipitation (Figure 9). In these materials, alkyl–alkyl and C_60_–C_60_ interactions determine the state, phase, morphology, and structure of the material, while the optoelectronic properties of C_60_ are retained. In particular, alkyl-fullerenes in the liquid state are prototypical functional molecular liquids [155] and have been developed into a variety of liquid materials by addition of alkyl chains to other functional, π-conjugated cores [156]. Control of the dielectric constant by fullerene bis-adducts has also been reported. Matsumoto et al. established a method for improving the dielectric constant of fullerene derivatives based on theoretical calculations and cheminformatics [157]. Improving the dielectric constant is important for the development of organic electronics. Linear C_70_ fullerene bis-adducts with two substituents were designed and characterized in thin-film devices. The fullerene bis-adduct had a relatively high dielectric constant and moderate electron mobility. This study demonstrates that high-throughput computational screening is effective for the design of fullerene bis-adducts with high dielectric constants.

**Figure 10:**
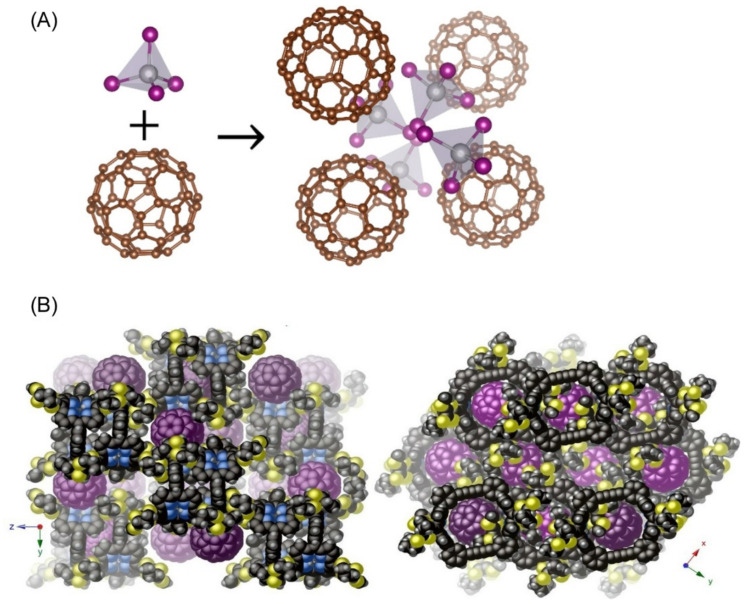
Fullerene-based molecular complex: (**A**) crystals from an icosahedral fullerene (C_60_) and tetrahedral SnI_4_ molecules; and (**B**) ball-and-socket type complex with C_60_ and extended tetrathiafulvalene-porphyrin scaffold. Reprinted with permission from [158]. Copyright 2020, American Chemical Society. Reprinted with permission from [159]. Copyright 2020, American Chemical Society.

The interaction of fullerenes with other molecules has also been reported to form characteristic assemblies. Straus and Cava reported an unusual example of chiral materials formed by self-assembly of achiral molecules [158], synthesizing C_60_(SnI_4_)_2_ crystals from an icosahedral fullerene (C_60_) and tetrahedral SnI_4_ molecules by spontaneous self-assembly (Figure 10A). The SnI_4_ tetrahedra forms a chiral, cubic 3-connected network of SrSi_2_-type using Sn atoms as templates. This result indicates the emergence of a self-assembled chiral material from the two most-symmetric molecules. In a broader sense, this result suggests that almost any molecule, nanocrystal, or artificial precursor can be considered as a building block for designing chiral assemblies. Kaur et al. reported that the extended tetrathiafulvalene-porphyrin scaffold acts as a ball-and-socket type receptor for C_60_ and C_70_ [159]. The supramolecular interactions of these fullerenes result in the formation of three-dimensional supramolecular organic frameworks composed of peapod-like linear assemblies in the solid state (Figure 10B). Thus, supramolecular organic frameworks fabricated via self-assembly may serve as tunable functional materials, the conductive properties of which can be tuned through the complementary geometry of their components and the choice of fullerenes.

**Figure 11:**
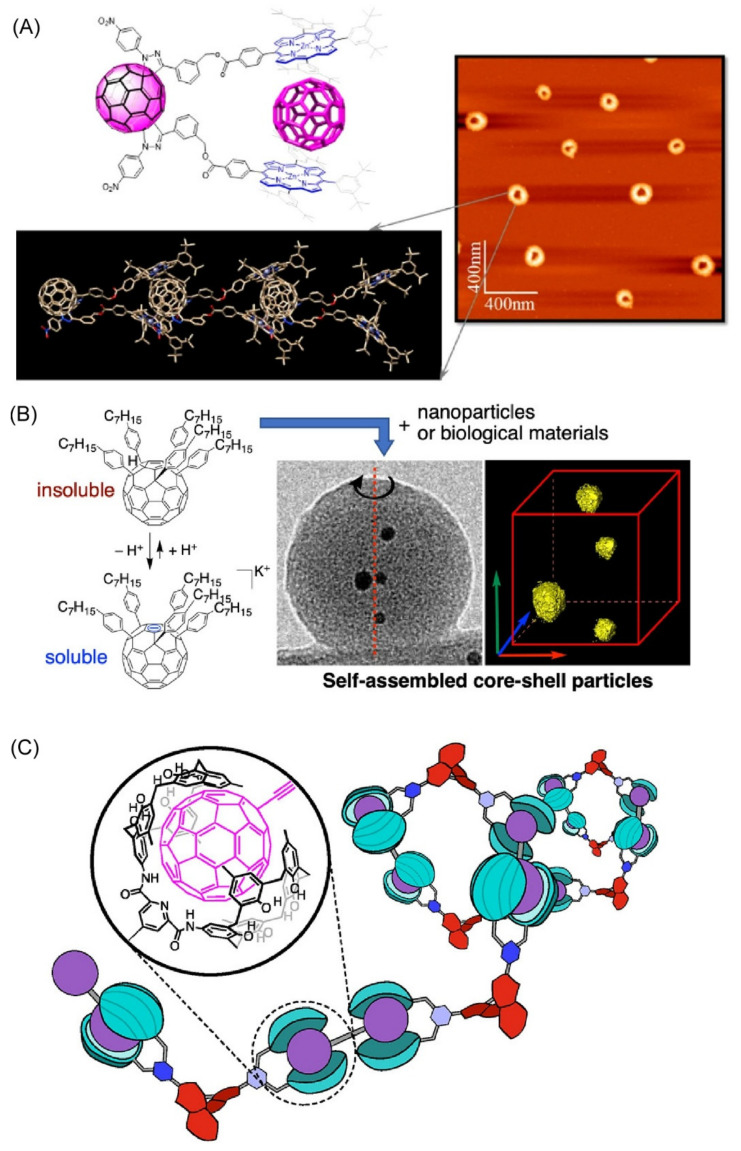
Fullerene-based assembly: (**A**) fullerene-bis-zinc-porphyrin electronic double adduct hierarchically structured to form doughnut-shaped aggregates; (**B**) protonated (4-heptylphenyl)_5_C_60_H self-assembly into fullerspheres (fullerene spheres); and (**C**) helically aligned fullerenes by supramolecular polymerization of a chiral ditopic tetrakiscalix[5]arene host and dumbbell-type fullerenes. Reproduced under terms of the CC-BY license [160]. Copyright 2021, American Chemical Society. Reprinted with permission from [161]. Copyright 2021, American Chemical Society. Reprinted with permission from [162]. Copyright 2021, American Chemical Society.

Caballero et al. synthesized a new fullerene-bis-zinc-porphyrin electronic double adduct [160]. The formation of supramolecular oligomers was confirmed, in which the two porphyrin arms encompass the fullerene cages of neighboring molecules. Furthermore, AFM observations showed that the initially formed worm-like oligomers hierarchically assembled to form doughnut-shaped aggregates (Figure 11A). The final supramolecular donut was found to have an estimated internal cavity of 23 nm, close to the size observed in photosynthetic antenna systems. This method of constructing giant, self-assembled donor–acceptor assemblies is important for nanoarchitectonics-type design of photoenergy harvesting systems. Harano and Nakamura et al. found that with gradual in situ protonation of (4-heptylphenyl)_5_C_60_-K^+^, the corresponding protonated molecules (4-heptylphenyl)_5_C_60_H self-assemble into fullerene spheres with uniform diameters ranging from 30 nm to 2.5 μm, which can be controlled by the pH of the buffer solution [161] (Figure 11B). They also successfully encapsulated aqueous solutions of organic dyes, inorganic nanoparticles, proteins, and viruses into fullerene spheres. The spheres are completely amorphous, thermally stable up to 300 °C under vacuum, and withstand electron irradiation. The spheres were found to be of unprecedented utility for electron tomography imaging of nanoparticles and biomaterials. Haino et al. successfully prepared helically aligned fullerenes by supramolecular polymerization of a chiral ditopic tetrakiscalix[5]arene host and dumbbell-type fullerenes [162] (Figure 11C). The molecular association of the chiral host with dumbbell-type fullerenes resulted in supramolecular polymers of considerable size in solution. The achiral dumbbell-type fullerenes exhibit circular dichroism in the π–π* transition band arising from the fullerene moiety due to supramolecular polymerization. The chiral twisted conformation of dumbbell-type fullerenes was also shown to be induced by supramolecular polymerization.

The development of functional materials based on fullerenes has also been extensively researched. Application research in energy-related fields is particularly active. Vinu and coworkers reported a nanotemplate method used to produce ordered mesoporous fullerene/carbon hybrids [163], where fullerene precursors were mixed with different amounts of sucrose in chloronaphthalene using SBA-15 mesoporous silica as a template. The synthesized materials are expected to be useful as electrodes for supercapacitors and lithium-ion batteries. For example, they showed much higher specific capacitance than that of activated carbon, multiwall carbon nanotubes, regular mesoporous carbon, and mesoporous C_60_. Carbon coating with mesoporous fullerenes is expected to be useful for energy storage devices because it facilitates electron transport between fullerene molecules and supports electrolyte access and diffusion due to its high specific surface area.

**Figure 12:**
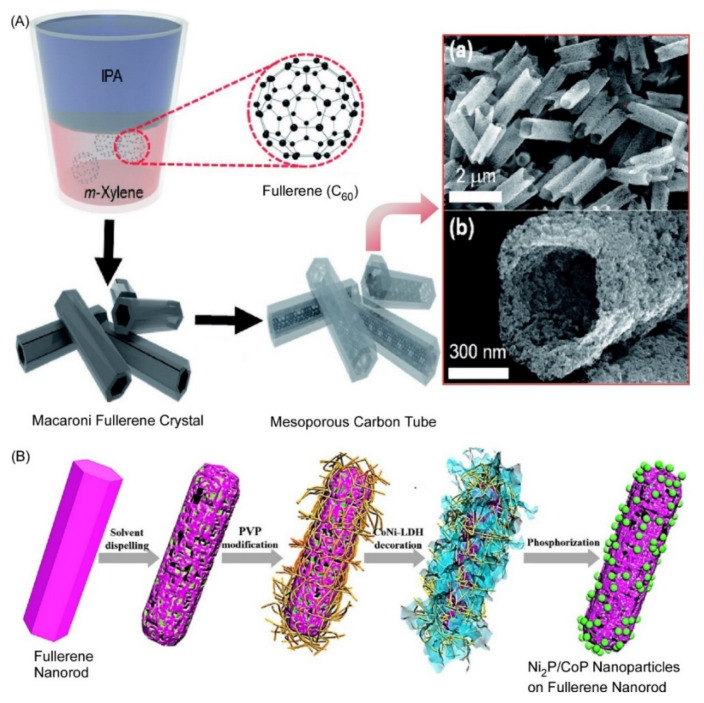
Modified fullerene crystals: (**A**) self-assembled macaroni fullerene C_60_ crystals and heat-treating conversion to mesoporous carbons tubes; and (**B**) hybrid nanostructures of Ni_2_P/CoP nanoparticles supported on porous fullerene nanorods as bifunctional electrocatalysts. Reprinted with permission from [164]. Copyright 2021, Chemical Society of Japan. Reprinted with permission from [165]. Copyright 2021, American Chemical Society.

Shrestha and coworkers prepared self-assembled macaroni fullerene C_60_ crystals with a uniform shape and narrow particle-size distribution at room temperature using a dynamic liquid–liquid interfacial precipitation method [164] (Figure 12A). By heat-treating the macaroni fullerene C_60_ crystals at high temperatures (900 °C), mesoporous carbon tubes were obtained that retained their initial morphology. This novel mesoporous carbon material exhibits excellent electrochemical supercapacitor performance due to its high surface area, large pore volume, and interconnected porous structure. The new mesoporous carbon material with π-electron moieties is expected to be an alternative electrode material for advanced supercapacitor devices. Feng, He, and coworkers developed hybrid nanostructures of Ni_2_P/CoP nanoparticles supported on porous fullerene nanorods as bifunctional electrocatalysts [165]. As shown in Figure 12B, fullerene nanorods were first heated to remove organic solvents in the crystal. Subsequently, they were surface-modified with polyvinylpyrrolidone and coated with cobalt–nickel layered double hydroxide. After phosphorization, these nanosheets were transformed into ultra-small Ni_2_P/CoP nanoparticles on the surface of porous N-doped fullerene nanorods. The hybrid nanostructures exhibited excellent electrocatalytic performance for water splitting in alkaline media due to the current-collecting effect of fullerene nanorods with Ni_2_P/CoP nanoparticles. This study opens the possibility of fullerene hybrid nanostructures in the field of energy conversion and storage.

**Figure 13:**
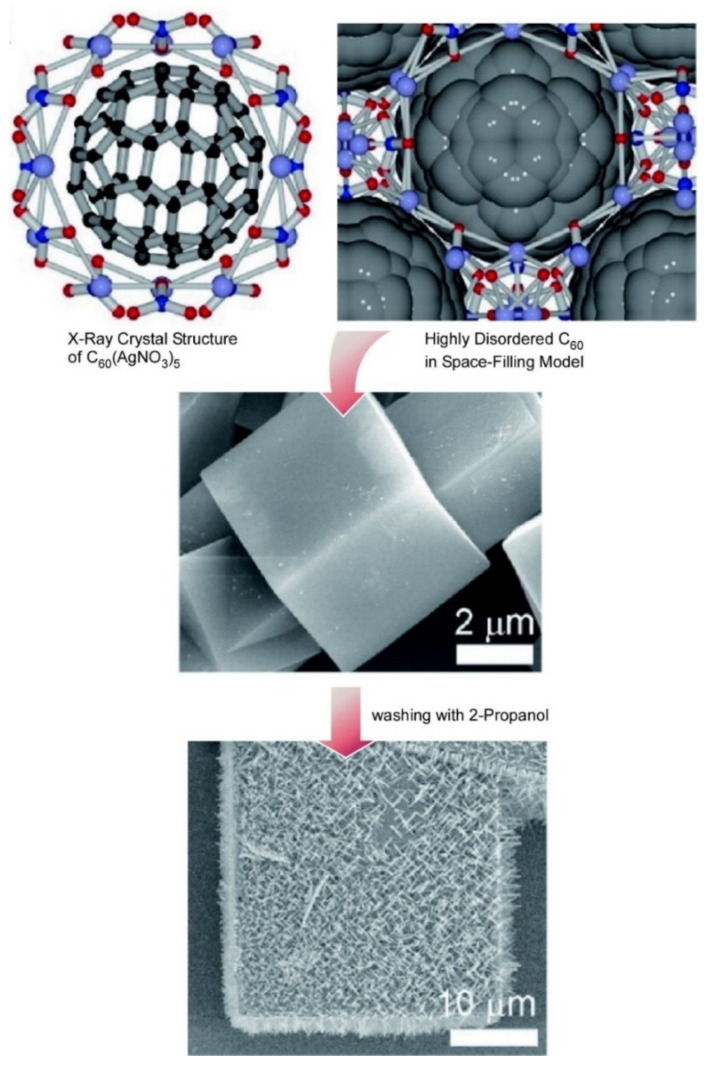
A cubic-type supramolecular fullerene C_60_-AgNO_3_ nanomaterial in which treatment with 2-propanol causes the growth of fine, needle-like C_60_ crystals on the cubic surface. Reprinted with permission from [167]. Copyright 2021, Chemical Society of Japan.

The development of high-quality organic nanoparticle inks is an important scientific challenge for the industrial production of solution-processed organic photovoltaic cells using environmentally friendly processing methods. Xie, Brabec, and coworkers developed a novel, robot-based method for automatically synthesizing poly(3-hexylthio-phene-2,5-diyl) and indene-C_60_ bisadduct nanoparticle inks using harmless alcohols. A high-throughput method is reported [166]. Robotic high-throughput organic nanoparticle ink preparation holds promise for environmentally friendly industrial production of organic photovoltaic cells. Fullerene assemblies and nanostructures have also been used for bio-related applications. Hill et al. investigated the sustained release of Ag from a cubic-type supramolecular fullerene C_60_-AgNO_3_ nanomaterial [167] (Figure 13). Treatment of this cubic C_60_(AgNO_3_)_5_ with 2-propanol causes the growth of fine, needle-like C_60_ crystals on the cubic surface, suggesting that Ag^+^ ions are continuously released from the complex when immersed in 2-propanol for a long time. It was also shown that AgNO_3_ precipitates on the insoluble C_60_ crystal framework concomitant with silver nanoparticles precipitating at the crystal–solution interface. Following the initial release of Ag^+^ (in the form of AgNO_3_), silver is reductively immobilized in the form of nanoparticles, resulting in longer-lasting bactericidal activity. The viability of bacterial colonies in aqueous media was significantly reduced. Cell-growth studies with HeLa cells revealed that C_60_(AgNO_3_)_5_ crystals exhibit antiproliferative properties in aqueous medium.

## 5. Biomedical Fullerene Nanoparticles

The above background descriptions impart several important messages: (i) advancement of nanoarchitectonics as a nanotechnology-based materials science is essential for innovation in functional material systems; (ii) nanostructured materials and their assemblies have made important contributions to basic biology and bio-related applications; and (iii) assembling nanomaterials from fullerene molecules as simple molecular units can manifest various properties and functions desirable in energy, environmental, and biological fields. As a natural culmination of this background information, research progress on biomedical fullerene nanoparticles is exemplified in this section. These examples present effective and important contributions of nanoarchitectonics based on fullerene as a simple unit being implemented in advanced and complicated biological applications.

### 5.1. Fullerene Nanoparticles for Biosensing and Fluorophore Reporting

Nanomaterials have been keenly discussed and studied as enhancers of biosensor sensitivity. Strategic incorporation of nanomaterials into biosensor structures often results in signal amplification arising from an abundance of active binding sites, outstanding electrocatalytic activity, and stable signals. Fullerenes (C_60_, C_70_) are particularly noteworthy for demonstrating outstanding inherent redox activity, which is absent in other materials and key to highly sensitive biosensors. These properties are often applied in conjunction with chemical modification of the fullerene structure to enhance analyte performance.

**Figure 14:**
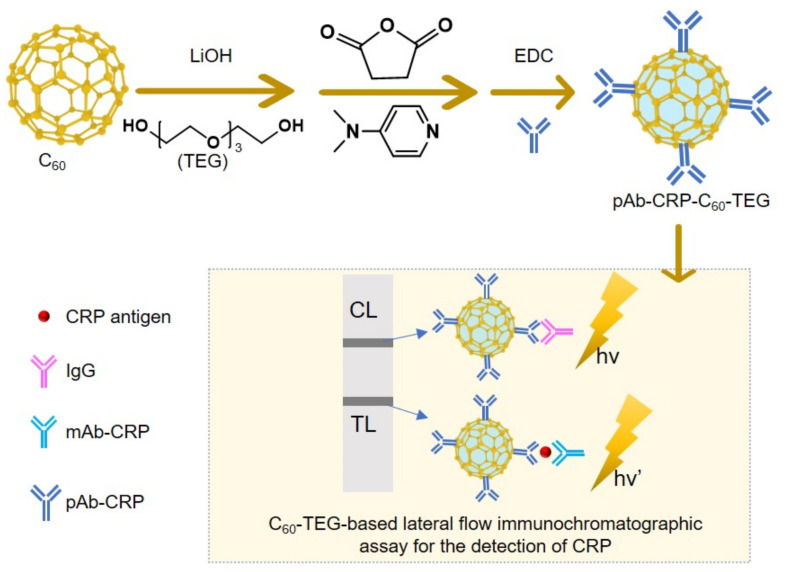
Schematic of the synthesis of pAb-CRP-C_60_-TEG for detecting CRP (C-reactive protein): CL, control line; TL, test line; mAb-CRP, monoclonal CRP antibody; pAb-CRP, polyclonal CRP antibody; and EDC, 1-ethyl-3-(3-dimethyllaminopropyl)-carbodiimide hydrochloride.

Lateral-flow immunochromatographic assays are a simple and rapid technique for detecting analytes (hormones, disease-related biomarkers, and toxins) in clinical, environmental, and food industry settings. Colloidal gold is the standard reporting material, but it has limited sensitivity and quantitative analysis capabilities. Other nanoparticles have demonstrated improvement in these areas, but challenges remain in material preparation, functionalization for efficient conjugation with target analytes, and optimization of sensing conditions. Kang, Jeong, and coworkers studied fluorescent fullerene nanoparticles conjugated with tetraethylene glycol (C_60_-TEG) that are easy to prepare with lithium-hydroxide catalyst at room temperature and provide distinct and controllable fluorescent signals (Figure 14) [168]. They report a polyclonal anti-CRP-conjugated C_60_-TEG (pAb-CRP-C_60_-TEG) fluorescent probe for highly sensitive, rapid, and quantitative lateral-flow immunochromatographic assay analysis of C-reactive protein (CRP) in serum. CRP is a critical marker for inflammation and cardiovascular disease. The probe was simply prepared by carboxylation of C_60_-TEG followed by 1-ethyl-3-(3-dimethyllaminopropyl)-carbodiimide hydrochloride coupling. A lateral-flow strip design incorporating the probe demonstrated a dynamic range of 0.1–10 ng/mL CRP, in which fluorescence signal ratio increased with CRP concentration. Preparation of pAb-CRP-C60-TEG was simple, and it successfully detected a wide range of CRP concentrations; C_60_-TEG-based lateral-flow immunochromatographic assay has strong potential for immunoassays.

**Figure 15:**
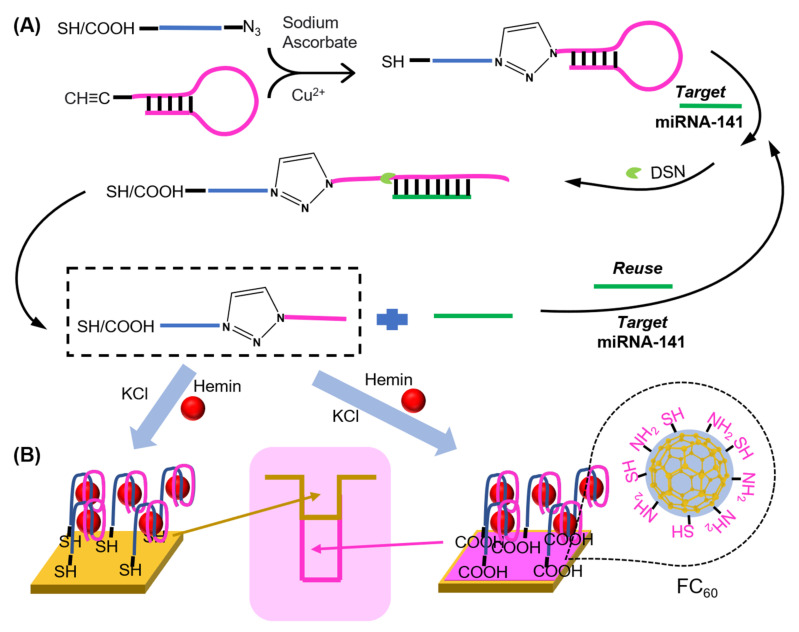
Schematic diagram of (**A**) EATR (enzyme-assisted target recycling) and (**B**) FC_60_ dual-amplification strategy for an electrochemical biosensor: DSN, duplex-specific nuclease; and FC_60_, amino and thiol group multi-functionalized fullerene nanoparticles.

Quantitative expression profiles of microRNAs (miRNAs) are potent biomarkers of cancer occurrence and progression. Due to their small size, low abundance, and sequence similarity, ultrasensitive detection and specific analysis remains challenging. In DNA nanotechnology, binding DNA to nanoparticles produces stable and amplified signals; copper (I)-catalyzed azide-alkyne cycloaddition is an outstanding click reaction with high yield, mild reaction conditions, and extremely high compatibility with functional groups. Further amplification can be achieved with carbon nanomaterials due to their notable electronic properties and potential for precise chemical modification. Zhou, Zhang, and coworkers introduced a dual-amplified strategy integrating click-chemistry-mediated enzyme-assisted target recycling with amino and thiol group multi-functionalized fullerene nanoparticles (Figure 15) [169]. The copper (I)-catalyzed azide-alkyne cycloaddition reaction effectively avoided unnecessary ion-bridge connection of nucleic acid, reducing false positives. The resultant DNA hairpin loop exhibited high specificity for the target miRNA (MiRNA-144). This yielded strong amplification during enzyme-assisted target recycling, which was further amplified electrochemically by fullerene nanoparticles immobilized on the Au electrode surface. The dual-amplification strategy exhibited excellent discrimination and higher analytical performance, forming the basis of an easily operated, high sensitivity, high selectivity, and more universally applicable biosensor platform.

**Figure 16:**
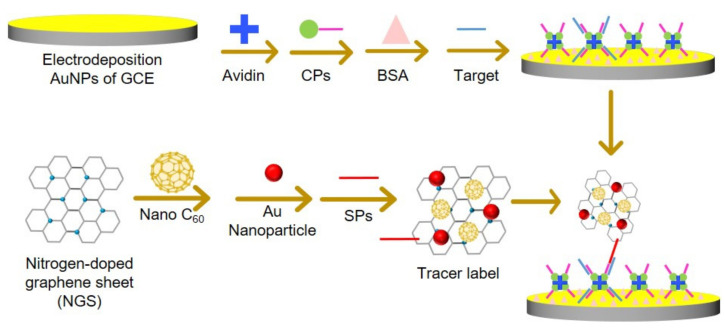
Schematic diagram of the synthesis of Au-nano C_60_/NGS-based electrochemical biosensor for amplifying the detection of target DNA: GCE, glassy carbon electrode; BSA, bovine serum albumin; CPs, GGTGAGGTCTAAAAA-biotin; Target, AGACCTCACCTATGTGTCGA; and SPs, SH-(CH_2_)_6_-AAAAATCGACACATA.

Tuberculosis is caused by *Myobacterium tuberculosis* (MTB). Targeting IS6110, an insertion element unique to MTB, as a detection marker, Guo and coworkers developed an electrochemical biosensor for MTB (Figure 16) [170]. The biosensor consists of nitrogen-doped graphene nanosheet as a matrix, which is simultaneously loaded with fullerene nanoparticles (nano-C_60_) and gold nanoparticles as a new signal tag for amplifying MTB DNA detection. Incorporating nitrogen-doped graphene nanosheet with nano-C_60_ combined a high-ratio surface, great conductivity, and abundant nitrogen atoms with outstanding redox activity. The abundance of nitrogen atoms enables immobilization of gold nanoparticles on the surface, which were labeled with signal probes via strong Au–S bonds (forming the tracer label) and enhanced electron transfer. The biosensor was also treated with tetraoctylammonium bromide to induce inherent electroactivity of the tracer label to output a current. The biosensor demonstrated synergistic effects of the nitrogen-doped graphene nanosheet, nano-C_60_, and gold nanoparticles, exhibiting a broad linear range for MTB detection from 10 fM to 10 nM. Furthermore, it distinguished the target DNA sequence from similar DNA sequences.

There is interest in fullerenes as fluorophores due to their highly symmetric spherical structure, but they exhibit extremely weak fluorescent emissions and inherent hydrophobicity. Functional modifications have been investigated but have not produced adequate quantum yield for practical applications. Ma and coworkers prepared fluorescent nanoparticles based on C_60_ by conjugating it with thiol-ene chemistry, followed by amidation with folic acid [171]. The fluorescent nanoparticles demonstrated a high quantum yield of 26% as well as stability in aqueous media and resistance to irradiation and pH. Fluorescent nanoparticles detected folic acid with a limit of detection of 0.24 µM. Since folic acid has a specific affinity with folate receptors, which are membrane glycoproteins expressed at high levels in many cancer cells, the group applied the fluorescent nanoparticles toward targeted imaging of cancer cells. The fluorescent nanoparticles were selectively internalized by folate-receptor-overexpressing cancer cells (HELA and U87) over noncancerous (COS-7) cells.

### 5.2. Fullerene Nanoparticles as Gene Delivery Vehicles

Fullerene nanoparticles are characterized by their spherical structures and strong hydrophobicity. However, chemical modifications have been used to create water-soluble fullerene derivatives adorned with hydrophilic moieties such as amino, carboxyl, and hydroxyl residues, which greatly improves biocompatibility. These same techniques can create amphipathic fullerenes, which are chemically modified with hydrophilic sidechains, offering great potential for gene delivery because they effectively form complexes with DNA. Furthermore, fullerene molecules have a small diameter of 1 nm, making them suitable for bypassing various barriers in the body.

**Figure 17:**
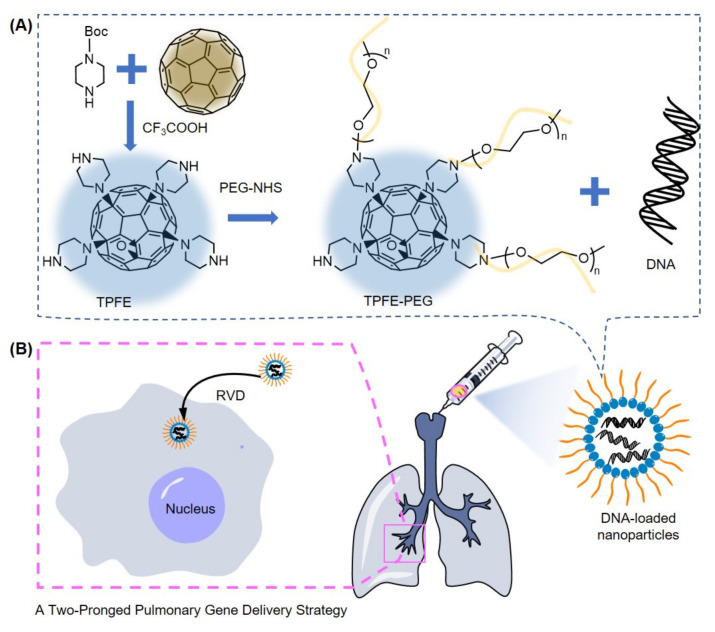
(**A**) Synthesis of Tetra(piperazino)fullerene (TPFE) and TPFE-PEG. (**B**) DNA-loaded nanoparticles (NPs) self-assembled by TPFE-PEG and DNA provided highly efficient transgene expression in lung parenchymal cells via the osmotically driven regulatory volume decrease (RVD) mechanism.

Shu, Suk, and coworkers investigate localized gene therapy via inhalation to treat refractory lung diseases (Figure 17) [172]. This is advantageous in mitigating risks of systemic adverse effects caused by extra-pulmonary gene transfer. However, airways are covered with highly adhesive and nano-porous mucus that traps and removes exogenous material, and alveolar sacs are inhabited by alveolar macrophages; together, they form challenging biological barriers to efficient delivery to the targeted lung parenchymal cells. Tetra(piperazino) fullerene epoxide (TPFE) has been demonstrated as an effective transfer agent in the liver and spleen, providing robust DNA compaction, endocytic cellular uptake, protection against endosomal nuclease, and cytoplasmic DNA release. However, its cationic and hydrophobic characteristics make it susceptible to mucus entrapment and macrophage uptake in the lung. This study conjugated polyethylene glycol (PEG) chains to TPFE’s piperazine components to form a non-adhesive surface coating that evades defense mechanisms, increasing probability of gene delivery. To address PEGylation’s effects on nanoparticle interactions, which may include reduced endocytosis, the group used a hypotonic vehicle (ultrapure water) to modulate osmolality through regulatory volume decrease. This mechanism uses hypotonic cell swelling to trigger fusion of intracellular vesicles into the plasma membrane; to achieve homeostasis, the cell releases ions and water, and it internalizes part of the plasma membrane to regenerate vesicles. The transfer agent is endocytosed during this process. Whereas the TPFE transfer agent significantly aggregated in mucin solution within 15 min, TPFE-PEG had unchanged hydrodynamic diameters upon incubation in mucin for an hour. This suggests that PEG efficiently prevents adhesive interactions with mucus. Nanoparticles were loaded with plasmid DNA encoding fluorescent Zsgreen protein (pZG) for visualization. In vitro assessment of phagocytosis by mouse alveolar macrophages indicated ~80% uptake of pZG/TPFE and ~20% uptake of pZG/TPFE-PEG. Intratracheal administration of pZG/TPFE-PEG to mice mediated widespread and uniform transgene expression in airways and alveolar sacs with >65% coverage, compared to <2% coverage with pZG-TPFE and naked pZG. The study also confirmed that the plasmid payload did not translocate to extra-pulmonary organs (liver, spleen, and kidney). The transfer agent was also tested on a mouse model with pathological mucus accumulation/stasis and chronic lung inflammation. With further delivery barriers, pZG/PTFE-PEG still provided ~35% coverage in the lungs. Administered in ultrapure water, transgene expression increased by an order of magnitude over identical nanoparticles administered in 0.9% saline. The result was verified to be attributable to regulatory volume decrease in a parallel experiment using an ion channel inhibitor. Hypotonic regulatory volume decrease plays a significant role in mediating efficient transgene expression. In this study, PEGylating the TPFE-based transfer agent overcame extracellular barriers, and a hypotonic vehicle facilitated endocytosis, resulting in synergistically enhanced pulmonary gene transfer.

### 5.3. Fullerene Nanoparticles as Radical Scavengers

Free radicals, also known as reactive oxygen species (ROS), are highly reactive chemicals formed from oxygen (e.g., O_2_^−^, H_2_O_2_, OH^−^, and NO). They are dangerous because they can cause irreversible damage to DNA and cells. Aside from external exposure, they exist naturally at background levels as part of the cellular respiration process. However, excessive ROS production under stress, usually by immune cells, has been pathologically linked to various inflammatory and autoimmune diseases. Fullerenes have been keenly considered for their therapeutic potential against such diseases. Due to their high degree of unsaturation arising from abundant π bonds, they effectively trap free radicals. As radical sponges, fullerenes may prove superior to natural ones (i.e., growth factors, cytokines, and enzymes) due to their long-lasting activity and cell-membrane-penetrating ability.

After sustaining an injury that cuts off blood supply to tissue (ischemia), restoration of blood flow could lead to further reperfusion injury. During reperfusion, activated neutrophils are attracted to the site of injury and release ROS. The ROS lead to apoptosis and damage DNA and mitochondria, resulting in additional loss of muscle function. Natural endogenous antioxidants can only neutralize a small amount of radicals. C_60_ fullerenes can efficiently capture free radicals with their abundant conjugated double bonds and low-energy LUMO orbitals. Ritter and coworkers investigated intramuscularly administered C_60_ fullerene aqueous solution for therapeutic and preventive value as an antioxidant [173]. The study found that C_60_ fullerene aqueous solution at a dose of 1 mg/kg reduces severity of ischemic damage in rat muscle soleus by 60–75%. Intramuscular injection produces a 52% therapeutic effect. Injection 1 h before injury had a 69% preventive effect, with biochemical markers of peroxidation and oxidative stress reduced by 45–60%, indicating promise for prophylactic application.

There are currently no safe and effective disease-modifying treatments for intervertebral disc degeneration. There is evidence that the complex immune response involving infiltration by immune cells and cytokine production at disc hernia sites is a prominent source of inflammation and pain. Dorn, Li, and coworkers targeted inflammatory cells for therapeutic development [174]. Formyl peptide receptor-1 is highly expressed in neutrophils, monocytes, and macrophages at an inflammatory site. The group previously demonstrated that cFlFlF peptide binds specifically to formyl peptide receptor-1 on activated macrophages and monocytes, which could be used to empower targeted drug delivery to disc hernia sites. Nanoparticle fullerene shows therapeutic potential for pathological conditions involving oxidative stress and inflammation. The group previously demonstrated therapeutic functions of fullerol in discogenic back/leg pain. In this study, a new formyl peptide receptor-1 targeted C_60_ nanoparticle (FT-C_60_) was developed to alleviate discogenic pain through systemic delivery (intravenous administration). The structure of FT-C_60_ uses functionalized C_60_ as the therapeutic moiety, and PEG and lysine as the linker and spacer, respectively, cFlFlF peptide as the targeting modality, and cyanine 5 fluorescence dye for detection. Electron paramagnetic resonance was used to evaluate radical scavenging capability. Both functionalized C_60_ and FT-C_60_ demonstrated strong radical scavenging; at 8 µM, FT-C_60_ effected 56% and 71% signal reduction for OH and O_2_^−^, respectively, making it approximately three times stronger than even functionalized C_60_. This strong antioxidative potential could be due to increased π bonds on the peptide that stabilized electrons. In vitro studies on macrophages (RAW 264.7) showed that FT-C_60_ significantly attenuated expression of inflammatory factors, including IL-6, TNF-α, IL-1, and COX-II. The protection effect is dose dependent. FT-C_60_ did not demonstrate cytotoxicity. In a mouse model, a single intravenous injection of FT-C_60_ (10 nmol/20 g) alleviated pain for up to 12 days post operation, as evaluated with the von Frey test. Ex vivo fluorescence imaging of the spine depicted accumulation of FT-C_60_ toward the inflammatory infiltration sites. Histological analysis corroborates that FT-C_60_ dramatically reduced inflammation at the injury site. This is the first demonstration of targeted delivery by systemic administration of nanoparticle therapeutics to treat degenerative disc diseases.

**Figure 18:**
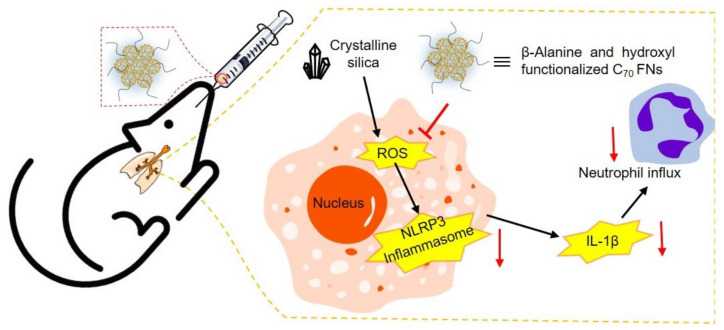
Proposed mechanism of fullerene nanoparticles (FNs) for the treatment of silicosis-associated pulmonary inflammation, crystalline silica significantly activates the intracellular ROS dependent pathway, which can be effectively attenuated by FNs. ROS: reactive oxygen species; NLRP3: NACHT, LRR, and PYD domains-containing protein 3.

Exposure to crystalline silica occurs in many occupations, and long-term inhalation leads to silicosis, a chronic, progressive fibrotic lung inflammation. Early intervention and control prevent destruction of lung structure and development into lung cancer, tuberculosis, and pulmonary disease. Silicosis onset begins with pulmonary neutrophilic inflammation; neutrophils produce ROS, which cause direct tissue injury, trigger apoptosis in alveolar macrophages, and promote secretion of cytokines that sustain and amplify inflammatory responses. Current therapeutics focus on blocking autoimmune functions or stimulating antioxidant expression rather than directly eliminating ROS. Fullerene nanoparticles have extraordinary ROS scavenging capability derived from their robust π-system that enables efficient electron capture; one fullerene nanoparticle molecule quenches multiple free radicals. Functionalized fullerene nanoparticle are also hydrophilic (convenient for pulmonary administration) and physiologically stable. In the study by Shu, Wang, and coworkers, β-alanine- and hydroxyl-functionalized C_70_ fullerene nanoparticles were synthesized via a liquid–liquid reaction and studied for potential therapeutic and preventive mechanisms (Figure 18) [175]. In vitro studies on macrophages (RAW 264.7) showed no cytotoxicity due to fullerene nanoparticles. When co-incubated with crystalline silica, fullerene nanoparticles reduced cell death in a dose-dependent manner. Electron paramagnetic resonance showed that 0.4 mgmL^−1^ fullerene nanoparticles could scavenge ~80% of hydroxy radicals in vitro. Crystalline silica significantly increases intracellular ROS levels, which can be effectively attenuated by fullerene nanoparticles. In vivo studies were performed on bronchoalveolar lavage fluid collected from a silicosis mouse model. The number of neutrophils significantly increased with intratracheally instilled crystalline silica. This number decreased in a dose-dependent manner after administration of fullerene nanoparticles. Expression levels of inflammatory cytokines (TNF-α, IL-1β, IL-6) collected from bronchoalveolar lavage fluid supernatant indicated significant upregulation due to exposure to silica, and dose-dependent downregulation with fullerene nanoparticle administration. Histological samples showed inflammatory cells infiltrated and damaged the alveolar structure. Mice treated with fullerene nanoparticles exhibited decreased inflammatory symptoms and recovery from lung structure damage. Assessment of ROS levels and related inflammatory signaling molecules in lung tissue showed upregulation with silica exposure and dose-dependent downregulation with fullerene nanoparticles, indicating that suppressed inflammation is associated with ROS scavenging. This study shows that pulmonary administration of fullerene nanoparticles could reduce oxidative stress induced by crystalline silica in lung tissues and further prevent downstream signaling activation of pulmonary inflammation.

The studies presented in this review are only a selection from recent research on biomedical fullerene nanoparticles. Nonetheless, the significant contributions of fullerene nanoparticles to practical biomedical applications should be recognized. Nanoarchitectonics based on a simple molecular unit of fullerene has the capacity to save lives.

## 6. Summary and Future Perspectives

The design, synthesis, and production of functional materials is key to solving a variety of current problems. Nanoarchitectonics is a new concept in which functional materials are created using a combination of processes from nanoscale units. It is expected to be a methodology for creating hierarchical and rationally functional structures. The methodology of nanoarchitectonics is reminiscent of processes and functions in biological systems. As described in this review article, control of nanostructures has had a major impact both in basic biology and medical applications. Therefore, the advantages of controlling nanostructures by means of nanoarchitectonics may be utilized to their highest potential in biomedical applications. As seen in this review article, the simple structure of the fullerene molecule determines the basic nanostructure of nanoparticles, which, in turn, effect complex biofunctions for active use in biomedical applications. This effectively embodies the molecule-to-material-to-bio nanoarchitectonics concept.

In this review paper, the biomedical effects of fullerenes were described in the context of fullerenes to nanoparticles. Fullerenes are zero-dimensional carbon allotropes that serve as a basic unit that can be transformed into one-dimensional structures [176,177], two-dimensional morphologies [178,179], three-dimensional motifs [180,181], and even more complex hierarchical structures [182,183]. In fact, some studies have reported the two-dimensional arrangement of one-dimensional structures, such as fullerene nanowhiskers that demonstrate control over cell growth and differentiation [184,185,186]. These fullerene materials in various structural motifs also demonstrate high potential for various biological and biomedical applications.

We have shown here that complex basic biology and biomedical applications can be derived from a very simple and basic molecule of fullerene (single element and zero-dimensional). Since this much can be done with such a basic structure, the diversity of functions that can be obtained is immeasurable if extended to molecules with more complex structures or with mixtures of different molecules. Applying a similar methodology to multicomponent systems would create unlimited possibilities for the fabrication of functional structures. Then, nanoarchitectonics may become a method for everything in materials science. Traditional experimental methodologies may prove too onerous to deal with complex multicomponent systems. Consequently, methods such as machine learning for material design [187,188,189] may prove powerful for exploring material structures and functions. Accordingly, developments such as a marriage of nanoarchitectonics and materials informatics would greatly advance the field [190]. The development of molecule-to-material-to-bio nanoarchitectonics, which embraces multicomponent and complex systems, will create a paradigm in material science that can respond effectively to fields of high social demands, such as biomedical fields. Positive signs can be seen in recent examples with application of nanostructured (nanoarchitectonics) materials for various fields as described in recent review articles [191,192,193,194,195]. Coupling of conceptual developments in molecule-to-material-to-bio nanoarchitectonics with demanded applications would make important contributions to create functional materials for social demands.

## Data Availability

Not applicable.
